# Human Dermcidin Protects Mice Against Hepatic Ischemia-Reperfusion–Induced Local and Remote Inflammatory Injury

**DOI:** 10.3389/fimmu.2021.821154

**Published:** 2022-01-14

**Authors:** Xiaoling Qiang, Jianhua Li, Shu Zhu, Mingzhu He, Weiqiang Chen, Yousef Al-Abed, Max Brenner, Kevin J. Tracey, Ping Wang, Haichao Wang

**Affiliations:** ^1^ The Feinstein Institutes for Medical Research, Northwell Health, Manhasset, NY, United States; ^2^ Donald and Barbara Zucker School of Medicine at Hofstra/Northwell, Hempstead, NY, United States; ^3^ TheraSource LLC, Manhasset, NY, United States

**Keywords:** dermcidin, chemokine, neutrophil, inflammation, tissue injury, EGFR

## Abstract

**Background:**

Hepatic ischemia and reperfusion (I/R) injury is commonly associated with surgical liver resection or transplantation, and represents a major cause of liver damage and graft failure. Currently, there are no effective therapies to prevent hepatic I/R injury other than ischemic preconditioning and some preventative strategies. Previously, we have revealed the anti-inflammatory activity of a sweat gland-derived peptide, dermcidin (DCD), in macrophage/monocyte cultures. Here, we sought to explore its therapeutic potential and protective mechanisms in a murine model of hepatic I/R.

**Methods:**

Male C57BL/6 mice were subjected to hepatic ischemia by clamping the hepatic artery and portal vein for 60 min, which was then removed to initiate reperfusion. At the beginning of reperfusion, 0.2 ml saline control or solution of DCD (0.5 mg/kg BW) or DCD-C34S analog (0.25 or 0.5 mg/kg BW) containing a Cys (C)→Ser (S) substitution at residue 34 was injected *via* the internal jugular vein. For survival experiments, mice were subjected to additional resection to remove non-ischemic liver lobes, and animal survival was monitored for 10 days. For mechanistic studies, blood and tissue samples were collected at 24 h after the onset of reperfusion, and subjected to measurements of various markers of inflammation and tissue injury by real-time RT-PCR, immunoassays, and histological analysis.

**Results:**

Recombinant DCD or DCD-C34S analog conferred a significant protection against lethal hepatic I/R when given intravenously at the beginning of reperfusion. This protection was associated with a significant reduction in hepatic injury, neutrophilic CXC chemokine (Mip-2) expression, neutrophil infiltration, and associated inflammation. Furthermore, the administration of DCD also resulted in a significant attenuation of remote lung inflammatory injury. Mechanistically, DCD interacted with epidermal growth factor receptor (EGFR), a key regulator of liver inflammation, and significantly inhibited hepatic I/R-induced phosphorylation of EGFR as well as a downstream signaling molecule, protein kinase B (AKT). The suppression of EGFR expression by transducing Egfr-specific shRNA plasmid into macrophages abrogated the DCD-mediated inhibition of nitric oxide (NO) production induced by a damage-associated molecular pattern (DAMP), cold-inducible RNA-binding protein, CIRP.

**Conclusions:**

The present study suggests that human DCD and its analog may be developed as novel therapeutics to attenuate hepatic I/R-induced inflammatory injury possibly by impairing EGFR signaling.

## Introduction

Hepatic ischemia and reperfusion (I/R) injury is an unavoidable consequence of circulatory shock, liver resection and transplantation, and represents a major cause of post-operative hepatic dysfunction, multiple organ failure, and even morbidity (1). It begins with an initial generation of reactive oxygen and nitrogen species (ROS and RNS) by liver macrophages (i.e., Kupffer cells) within a few hours of reperfusion. This process exerts direct but moderate hepatic injury, and facilitates the infiltration of neutrophils, which release more ROS and proteases to exacerbate a cascade of inflammatory injury (2, 3). Furthermore, the generation of ROS and RNS also leads to hepatocellular death (2, 4) and consequent release of damage-associated molecular patterns (DAMPs) such as cold-inducible RNA-binding protein (CIRP) (5) and high mobility group box 1 (HMGB1) (6, 7). We and others have shown that HMGB1 (8) and CIRP (9) exacerbate liver damage in animal models of hepatic I/R, as these DAMPs can amplify a cascade of oxidative and inflammatory responses during a late stage of reperfusion (1, 10). Despite on-going efforts in developing various pharmacological modalities to reduce hepatic I/R injury, there is still an unmet need for effective therapies (11). It is thus important to develop novel strategies to modulate local and remote inflammatory responses in patients who undergo liver transplantation, surgical resections or traumatic liver injury.

Human skin contains sweat glands that can secrete a wide array of antimicrobial peptides to restrain the growth of microbial pathogens. For instance, during rigorous exercise, an antimicrobial peptide, dermcidin (DCD), is secreted by the sweat glands onto the skin surface even in the absence of inflammatory stimuli (12, 13). It was believed that the salty and slightly acidic sweat facilitated the formation of DCD channels capable of perforating bacterial membranes to instill microbial killing (14–16). After its secretion, the full-length DCD precursor (residue 20-110) can be processed by unknown proteases into shorter peptides with anti-oxidant (14, 17) or antimicrobial activities (12, 18–20). In addition to sweat glands, some immune cells (e.g., monocytes) also express DCD after viral infections (21). Recently, we demonstrated that the full-length DCD precursor attenuated the production of nitric oxide (NO) and chemokines (e.g., GRO and MCP-3) induced either by pathogen-associated molecular patterns (PAMPs such as LPS) or damage-associated molecular patterns (DAMPs such as HMGB1 and CIRP) (22). It was previously unknown, however, how DCD or analogs affects innate immune responses to sterile injury in preclinical settings. In the present study, we sought to explore the therapeutic potential and protective mechanisms of DCD and analog in a murine model of hepatic I/R injury.

## Materials and Methods

### Materials

Dulbecco’s Modified Eagle’s Medium (DMEM, Cat. No. 11995-065), fetal bovine serum (FBS, Cat. No. 26140079) and penicillin/streptomycin (Cat. No. 15140-122) were purchased from Invitrogen (Grand Island, New York). Recombinant mouse CIRP was expressed in *E. coli*, and purified to remove contaminating endotoxins by Triton X-114 extraction as previously described (5). Recombinant human dermcidin (corresponding to residue 20-100, without the N-terminal 19-aa leader sequence) was expressed in *E. coli* BL21 (DE3) pLysS cells as previously described (22). To explore the therapeutic potential of DCD derivatives, an analog of DCD containing a Cys (C)→Ser (S) substitution at residue 34 (DCD-C34S) was also produced in *E. coli* BL21 (DE3) pLysS and purified to homogeneity using similar procedures. Recombinant DCD or DCD-C34S analog was purified by Triton X-114 extraction to remove contaminating endotoxins as previously described (22).

Adult male C57BL/6 mice (8-9 weeks old, 20-25 g body weight) were purchased from Charles River Laboratories (Wilmington, MA), and housed in a temperature-controlled room on a 12-h light-dark cycle. Mice were fed with a standard mouse chow diet, and acclimated to the environment for 5-7 days before usage. Every attempt was made to limit the number of animals used in the present study according to the ARRIVE guidelines for reducing the number of animals in scientific research developed by the British National Centre for the Replacement, Refinement and Reduction of Animals in Research (NC3Rs). Additionally, all experiments were performed in accordance with policies of the United States’ National Institutes of Health and the Guide for the Care and Use of Laboratory Animals, and approved by the Institutional Animal Care and Use Committee (IACUC) of the Feinstein Institutes for Medical Research.

### Animal Model of Hepatic I/R

Hepatic I/R was performed in male C57BL/6 mice (8-9 weeks old, 20-25 g) as described previously (9, 23). Animals were anesthetized by inhalation of 2-4% isoflurane, the ventral abdomen was shaved and alternately disinfected with Betadine and 70% alcohol. The animals were placed on a heating pad connected to an indwelling rectal thermometer to maintain core body temperatures of 35°C. A 1-1.5 cm midline incision was performed to expose the liver and the ligamentous attachments connecting the liver, and the diaphragm and abdominal wall were then divided to expose hepatic artery and portal vein. A vascular micro-clip was placed across the hilum containing the left and median lobes of the liver for 60 min to produce 70% ischemia, which was confirmed by the color change. Reperfusion was initiated by the release of the clamp before closing the abdomen wound with staples (wound clips). Given the undesired effects of opioid analgesics on hosts’ inflammatory responses to hepatic I/R (24–26) and other insults (27, 28), we have elected to use only a single dose of buprenorphine (0.05 mg/kg, subcutaneously) around the incision site to alleviate the immediate pain caused by surgical laparotomy. Afterwards, all animals were resuscitated by a subcutaneous injection of sterile saline solution (20 ml/kg). Blood and liver tissues were collected at 24 h after the onset of reperfusion. A portion of the left lobe of the liver was preserved in 10% formalin for histopathological analysis, and the remaining tissue was stored at -80°C for quantitative analysis.

For the survival study, the remaining 30% of non-ischemic liver was resected with electrocautery at the start of reperfusion, so the hepatic I/R could cause animal lethality in this model (9, 23). Afterwards, control vehicle (saline) or solution containing purified DCD or DCD-C34S analog were given to animals intravenously *via* internal jugular vein at the beginning of reperfusion, and animals were monitored for survival for up to 10 days.

### Measurement of Organ Injury Markers

Blood samples were harvested at 24 h post the onset of reperfusion, and centrifuged at 3000 x g for 10 min to collect serum. Serum levels of liver injury markers such as alanine aminotransferase (ALT, Cat. No. 7526), aspartate aminotransferase (AST, Cat. No. 7561) and lactate dehydrogenase (LDH, Cat. No. 7572) were determined using specific colorimetric enzymatic assays (Pointe Scientific, Canton, MI) as per the manufacturer’s instructions.

### Measurement of Cytokines

Mice were sacrificed at 24 h post the onset of reperfusion, and liver tissues were collected to measure TNF, IL-6, and IL-1β levels using commercial enzyme-linked immunosorbent assay (ELISA) kits (BioSource International, Camarillo, CA) as per the manufacturer’s instruction.

### Measurement of Nitric Oxide

The levels of nitric oxide (NO) in the liver tissue lysate or culture medium were determined by measuring the NO^2−^ production based on the colorimetric Griess reaction (29, 30). The NO^2−^ concentrations were deduced with reference to standard curves of sodium nitrite generated at various dilutions.

### Assessment of Hepatic Granulocyte Myeloperoxidase

Ischemic liver tissue (100 mg) was weighed and homogenized by sonication in 1 ml of potassium phosphate buffer containing 0.5% hexadecyltrimethylammonium bromide. Two freeze-thaw cycles were performed and then samples were centrifuged to collect the supernatant. Neutrophil accumulation within the liver was then estimated using the myeloperoxidase (MPO) activity assay based on the MPO-catalyzed chemical reaction that converted O-dianisidine dihydrochloride and H_2_O_2_ into colorimetric product detectable by light absorbance at 460 nm over a period of 5 min. The MPO levels were expressed as units per gram of tissue per minute.

### Immunohistochemical Staining of Gr-1–Positive Neutrophils

Paraffin-embedded liver or lung tissue sections were dewaxed in xylene, and rehydrated in a graded series of ethanol. Briefly, the slides were heated at 95°C for 30 min in 0.92% citric acid buffer (Vector Laboratories, Burlingame, CA). After cooling, the slides were incubated with 2% H_2_O_2_/60% methanol, and blocked in Tris-buffered saline containing 10% rabbit serum. The anti-Gr-1 antibody (BioLegend, San Diego, CA) was applied and incubated overnight. The detection was carried out using the NovaRED substrate of an immunohistochemistry kit (Vector Laboratories). Gr-1 positive neutrophils were counted under a high-power field microscopy (HPF; ×200) of 4 randomly selected areas. The number of neutrophils per HPF was determined by averaging the counts of 4 HPFs.

### Measurement of Cytokine mRNA by Real-Time RT-PCR

Total RNA was extracted from ischemic liver tissue using TRIzol Reagent Kit as per the manufacturer’s instructions (Invitrogen, Thermo Fisher Scientific Inc.), and was reverse-transcribed into the first-strand cDNA using the RevertAid™ First Strand cDNA Synthesis Kit (Applied Biosystems, Thermo Fisher Scientific Inc.). Following reverse transcription, a panel of primers for murine iNOS, Mip-2, and β-actin were used to quantify the mRNA expression levels of respective genes using an ABI 7900HT Fast Real-time PCR system (Applied Biosystems, Foster City, CA). The sequence of primers for this study is listed as follows: mouse iNOS, 5′-GGCAAACCCAAGGTCTACGTT-3′ (forward) and 5′-GAGCACGCTGAGTACCTCATTG-3′ (reverse); mouse Mip-2, 5′-CCCTGGTTCAGAAAATCATCCA-3′ (forward) and 5′-GCTCCTCCTTTCCAGGTCAGT-3′ (reverse); mouse β-actin, 5′-CGTGAAAAGATGACCCAGATCA-3′ (forward) and 5′-TGGTACGACCAGAGGCATACAG-3′ (reverse). Amplification was performed using the RT² SYBR Green ROX qPCR Mastermix under the following conditions: 95°C 10’; followed by 40 cycles of 95°C for 15” and 60°C for 1’. The relative mRNA expression level for each gene was calculated using the following formula: ΔΔC expression = 2–ΔΔCt, where ΔΔCt = ΔCt (treated group) – ΔCt (control group), ΔCt = Ct (target gene)–Ct (β-actin), and Ct = cycle at which the threshold was reached. The relative abundance of each mRNA expression in the sham control group was set as an arbitrary unit of 1, and the gene expression in treated groups was presented as folds of change in comparison to the sham group after normalization to β-actin.

### Histological Evaluation of Liver and Lung Injury

The left lobe of liver and lung samples were harvested at 24 h post reperfusion, and fixed in 10% buffered formalin before being embedded in paraffin. Paraffin-embedded tissues were cut into 5-μm sections, stained with hematoxylin-eosin and examined under light microscopy. As previously described (23), liver parenchymal injury was assessed in a blinded fashion by the sum of three different Suzuki scores ranging from 0-4 for sinusoidal congestion, vacuolization of hepatocyte cytoplasm, and parenchymal necrosis (23). Scores for each finding ranged from 0 to 4, with a maximum possible score of 12. The percent necrotic area was estimated by randomly evaluating 4 low-power fields (x100) of each hematoxylin-eosin–stained section using software Image J. Similarly, lung tissue sections were scored in a blinded fashion using a semi-quantitative scoring system developed by the American Thoracic Society as previously described by Matute-Bello et al. (31). As previously described (32), histological lung injury was scored based on alveolar septal thickening, as well as the presence of infiltrated inflammatory cells in the alveolar and interstitial spaces, and the presence of hyaline membranes and proteinaceous debris within airspaces according to the following definition: 0, no injury; 1, moderate injury; 2, severe injury. Using a weighted equation with a maximum score of 100 per field, the parameter scores were calculated and averaged as the final lung injury score in each experimental group.

### Western Blot Analysis

Liver tissue samples were homogenized in lysis buffer (10 mM Tris-HCl pH 7.5 with 1% Triton X-100, 1 mM EDTA/EGTA, 2 mM Na3VO4, 0.2 mM PMSF) containing a protease inhibitor cocktail (Roche, Indianapolis, Indian). Protein concentrations were determined using DC protein assay (Bio-Rad, Hercules, CA). Equal amount of tissue homogenates or macrophage cell lysates were fractionated on SDS-PAGE and transferred to nitrocellulose membrane. The membrane was incubated with antibodies to phosphor-EGFR/EGFR or phosphor-AKT/AKT (Cell Signaling, Danvers, MA), followed by secondary antibody-horseradish peroxidase conjugate (LI-COR Biosciences). Visualization and quantification was carried out with the LI-COR Odyssey^®^ scanner and software (LI-COR Biosciences).

### Surface Plasmon Resonance Analysis of DCD-EGFR Interaction

We used both the newly developed Nicoya Lifesciences Open Surface Plasmon Resonance (OpenSPR) and the traditional Biacore SPR techniques to evaluate the possible DCD/EGFR interaction as previously described (32–34). In contrast to the traditional Biacore SPR that uses a continuous film of gold to detect the angle change of re-emitted light when the surface plasmon wave interacts with a local particle, the Open SPR uses gold nanoparticles to detect small changes in the wavelength of conjugated adsorbing molecules after interacting with local ligand. For the Nicoya OpenSPR, highly purified recombinant DCD or DCD-C34S with 6×His Tag was immobilized on nitrilotriacetic acid (NTA) sensor chip (Cat. # SEN-Au-100-10-NTA), and recombinant EGFR corresponding to the extracellular domain (residue 25-645, Cat. No. AT#230-30016-250, RayBiotech) was applied at three different concentrations. The response signals were recorded over time, and the equilibrium dissociation constant (K_D_) was estimated using the Trace Drawer Kinetic Data Analysis v.1.6.1 (Nicoya Lifesciences). For the traditional SPR, highly purified recombinant DCD was immobilized on CM5 chip (GE Healthcare), and the recombinant EGFR was applied at 5 different concentration using a Biacore T200 instrument (GE Healthcare). The K_D_ was determined using the Biacore evaluation software 2.0 (GE Healthcare) supposing a 1:1 binding ratio.

### Knockdown of EGFR Expression in Macrophage Cultures

Murine macrophage-like RAW 264.7 cells were obtained from ATCC (ATCC, Rockville, MD), and cultured in DMEM (Invitrogen, Grand Island, New York) or RPMI 1640 (Invitrogen) supplemented with 10% heat-inactivated FBS, 1% penicillin/streptomycin and 2 mM glutamine at 37°C with 5% CO_2_. To evaluate the possible role of EGFR in the regulation of DCD-mediated anti-inflammatory action, we transfected RAW 264.7 cells with lentivirus particles expressing Egfr-specific shRNAs to down-regulate EGFR expression. The use of lentivirus to express Egfr-specific shRNA in macrophages has been approved by Institutional Biosafety Committee under an IBC Registration #R-2017-007, entitled “Role of EGFR in the regulation of dermcidin-mediated anti-inflammatory action”. The lentivirus particles were produced in human kidney Lenti-X™ 293T cells (Clontech) by co-transfer pLKO1 vector plasmid or EGFR shRNA-expression plasmid (Sigma Aldrich) together with packaging plasmids pCMV-dR8.2 dvpr and pCMV-VSV-G (Addgene) using Feugene 6 transfection agent (Promega). Lentiviral particles encoding pLK01 vector or EGFR shRNA were harvested from medium at 48 and 72 h post-transfection, and used to transduce murine macrophage-like RAW 264.7 cells for 8 h following standard procedures. Puromycin was added to fresh culture medium every 2-3 days until resistant colonies were identified. Afterwards, adherent macrophages were gently washed with, and cultured in, DMEM before stimulation with recombinant mouse CIRP (2.0 μg/ml) in the absence or presence of recombinant DCD for 16 h. Subsequently, the cell-conditioned culture media were analyzed for levels of nitric oxide (NO) by the Griess Reaction as previously described (35, 36).

### Statistical Analysis

All experimental data were assessed for normality by using the Shapiro-Wilk test before conducting appropriate statistical tests. For comparison among multiple groups with skewed (non-normal) distribution, the statistical differences were evaluated by using the non-parametric Kruskal-Wallis ANOVA test followed by the Dunn’s *post hoc* test. For comparison among multiple groups with normal data distribution, the differences were analyzed by using the parametric one-way analyses of variance (ANOVA) followed by Fisher Least Significant Difference (LSD) *post hoc* test. For survival studies, the Kaplan-Meier method was used to compare the differences in mortality rates between groups with the nonparametric log-rank *post hoc* test. A *P* value < 0.05 was considered statistically significant.

## Results

### Full-length DCD and DCD-C34S Analog Conferred a Significant Protection Against Lethal Hepatic I/R

**Figure 1:**
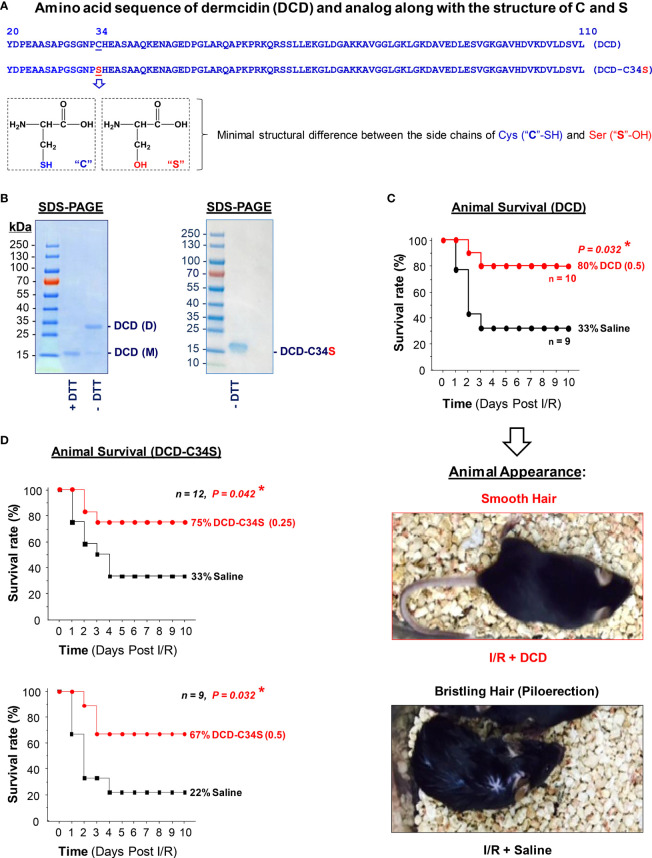
Recombinant dermcidin (DCD) and DCD-C34S analog conferred significant protection against lethal hepatic ischemia-reperfusion injury. **(A)** Amino acid sequence of full length DCD precursor (residue 20-110) and a DCD-C34S analog containing a Cys **(C)**→Ser (S) substitution at residue 34. The structural difference between Cys **(C)** and Ser (S) was also noted. **(B)** SDS-PAGE analysis of recombinant DCD (residue 20-110) and DCD-C34S analog with an N-terminal 6×His tag. Recombinant DCD migrated as a 16-kDa monomer (M) in the presence of a reducing agent (DTT), but migrated as both a 16-kDa monomer (M) and 32-kDa dimer **(D)** in the absence of DTT, suggesting possible disulfide cross-linkage to form DCD dimers. In contrast, DCD-C34S analog migrated as a homogenous 16-kDa band even in the absence of DTT. **(C, D)** Highly purified DCD and DCD-C34S analog conferred significant protection against lethal hepatic ischemia-reperfusion injury. Male C57BL/6 mice were subjected to hepatic ischemia for 60 min to produce injury in 70% of the liver. At the beginning of the reperfusion, the remaining 30% of the non-ischemic liver portion was surgically resected, and 0.2 ml saline or solution containing DCD (0.5 mg/kg BW) or DCD-C34S (0.25 or 0.5 mg/kg BW) was injected *via* the internal jugular vein, and animals were monitored for survival for up to 10 days. The Kaplan-Meier method was used to compare the differences in mortality rates (with skewed distribution) between groups with the nonparametric log-rank *post hoc* test. *P value < 0.05 versus “saline” control group. Representative images showed the respective absence or presence of piloerection in the DCD treatment group (“I/R + DCD”) and saline control group (“I/R + Saline”) at 24 h post reperfusion.

As previously described (22), we generated full-length recombinant human DCD corresponding to residue 20-110 (without the signal leader peptide, residue 1-19, **Figure 1A**) in *E. coli*, and verified its purity by SDS-PAGE analysis (**Figure 1B**). Because DCD contains a Cys residue (**Figure 1A**), it could be oxidized to form an intermolecular disulfide bridge between two DCD molecules, thereby presenting DCD as a heterogeneous mixture of monomers and dimers with varying equilibrium. Consistent with previous reports (22, 37), recombinant DCD with a His-tag migrated as a 16-kDa band on SDS-PAGE gel in the presence of a reducing agent, dithiothreitol (“+DTT”) (**Figure 1B**, Left Panel), but migrated as 16-kDa and 32-kDa bands in the absence of DTT (“-DTT”), confirming that Cys (C)-containing DCD could form dimers through intermolecular disulfide cross-linking. Following extensive extraction with Triton X-114 to remove contaminating endotoxins, we tested the therapeutic efficacy of highly purified DCD in a murine model of hepatic I/R injury. Recombinant DCD conferred a significant protection against lethal hepatic I/R, increasing animal survival from 33% in the saline control to 80% in the DCD-treatment group (**Figure 1C**). Consistent with the improvement in animal survival, other pathological symptoms such as piloerection were similarly attenuated by DCD treatment (**Figure 1C**). Piloerection refers to the process of hair bristling due to involuntary contraction of piloerector muscles at the base of hair follicles, often as a reflexive response of the sympathetic nervous system to startling stimulus in an attempt to insulate hair layers to trap heat. It will thus be important to further elucidate the intricate mechanism underlying the DCD-mediated prevention of I/R-induced piloerection in future studies.

However, the development of protein therapeutics often requires the production of homogeneous peptides unable to form aggregates that could adversely trigger immunogenic responses or other side effects. Furthermore, DCD contains a single Cys residue (**Figure 1A**) that is unable to form intramolecular disulfide bridge to influence its own tertiary structure. Given the minimal structural difference between the side chains of Cys (-SH group) and Ser (-OH group, **Figure 1A**), we generated an analog of DCD containing a Cys (C)→Ser (S) substitution at residue 34 (DCD-C34S, **Figures 1A, B**) to prevent intermolecular disulfide cross-linking. Indeed, recombinant DCD-C34S migrated as a 16-kDa monomer in SDS-PAGE gel even in the absence of a reducing agent, DTT (**Figure 1B**, Right Panel). Similarly, this DCD-C34S analog significantly increased animal survival rates when given at an identical (0.5 mg/kg BW) or a lower (0.25 mg/kg BW; **Figure 1D**) doses, confirming that DCD and DCD-C34S analog confer similar protections against lethal hepatic I/R. It also supports a therapeutic potential for the homogenous DCD-C34S analog in preclinical settings.

### Intravenous DCD Administration Attenuated Hepatic I/R-Induced Liver Injury

**Figure 2:**
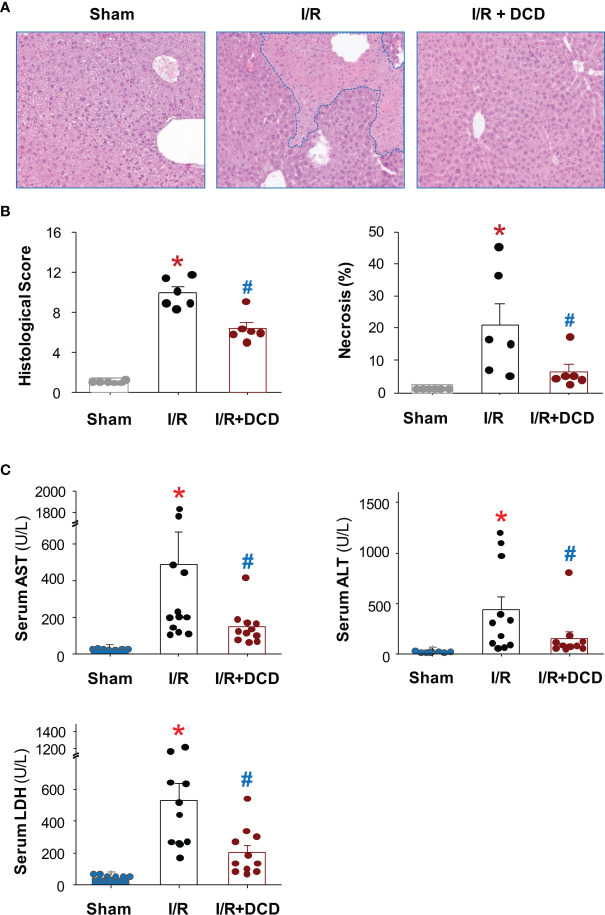
Recombinant DCD attenuated hepatic I/R injury. Male C57BL/6 mice were subjected to hepatic ischemia for 60 min, and treated with control vehicle or DCD solution (0.5 mg/kg) at the beginning of the reperfusion. At 24 h post the onset of reperfusion, the liver tissue was subjected to histological analysis using the Suzuki liver injury scores and % of necrosis (with skewed distribution, **(A, B)**, as well as biochemical assays of liver injury markers such as alanine aminotransferase (ALT), aspartate aminotransferase (AST) and lactate dehydrogenase (LDH) using commercial kits (with skewed distribution, **(C)**. Various liver injury scores and biomarkers were expressed as means ± SEM of 6 animals per group, and compared by Kruskal-Wallis ANOVA test followed by the Dunn’s *post hoc* test. *P < 0.05 vs. “Sham”; ^#^ *P* < 0.05 vs. “I/R” group.

To elucidate the mechanisms underlying DCD-mediated protection against lethal hepatic I/R, we examined the impact of DCD administration on hepatic I/R injury. At the dose that conferred protection against lethal hepatic I/R, DCD also significantly reduced hepatic I/R-induced liver injury (**Figure 2A**), as judged by the reduction in histological scores of hepatocellular necrosis, cytoplasmic vacuolization, sinusoidal congestion, and cellular infiltration at 24 h post the onset of reperfusion (**Figure 2B**). Consistently, blood levels of hepatic injury markers such as liver enzymes (AST and ALT) and LDH were also significantly reduced by DCD administration (**Figure 2C**), suggesting that DCD conferred significant protection against lethal hepatic I/R partly by attenuating I/R-induced liver injuries.

### Intravenous DCD Administration Inhibited Hepatic Neutrophil Infiltration and Inflammation

**Figure 3:**
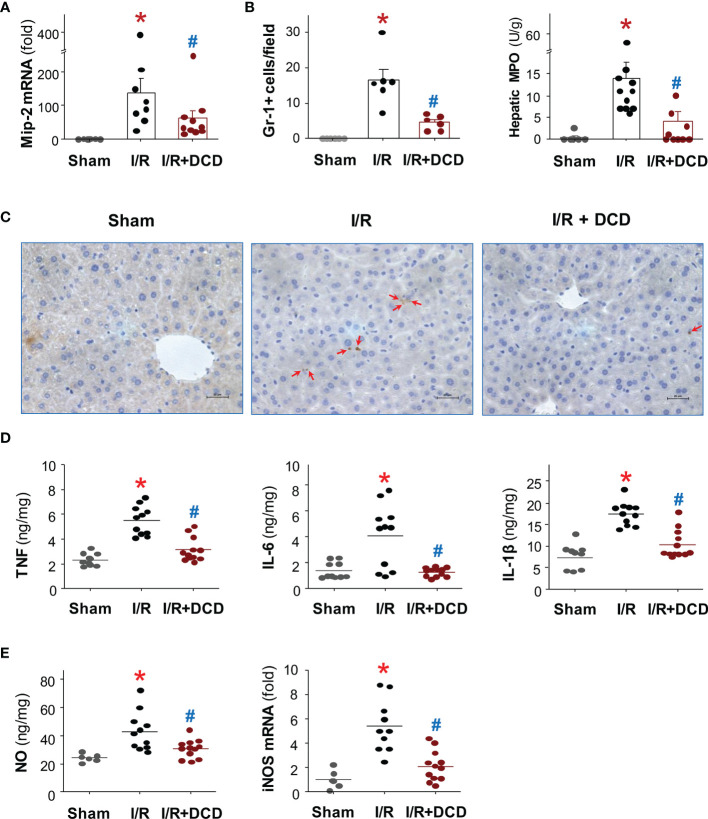
Recombinant DCD attenuated hepatic I/R-induced local inflammation. Male C57BL/6 mice were subjected to hepatic ischemia for 60 min, and treated with control vehicle or DCD solution (0.5 mg/kg) at the beginning of the reperfusion. At 24 h post the onset of reperfusion, the liver tissue was subjected to assays of Mip-2 mRNA expression **(A)**, neutrophil infiltration **(B, C)**, as well as production of proinflammatory cytokines **(D)** and nitric oxide (NO, **(E)**), as well as expression of inducible nitric oxide synthase (iNOS, **(E)**). Data were compared by parametric one-way ANOVA followed by the Fisher Least Significant Difference (LSD) *post hoc* test. *P < 0.05 versus “Sham”; ^#^ *P* < 0.05 versus “I/R” group.

To further explore the mechanisms underlying DCD-mediated protection, we further examined the effect of DCD administration on hepatic leukocyte infiltration and associated inflammation. The administration of DCD at the beginning of reperfusion resulted in a significant reduction in the expression of a neutrophilic CXC chemokine, macrophage inflammatory protein 2 (Mip-2/Cxcl2), at 24 h post reperfusion (**Figure 3A**). This suppression of Mip-2 expression was associated with a parallel reduction in the number of Gr-1-positive neutrophils infiltrated into the ischemic hepatic tissues (**Figure 3B**, Left Panel), as judged by immunohistological analysis of Gr-1-positive cells in the liver tissue (**Figure 3C**), as well as a biochemical assay of hepatic MPO enzyme activities (**Figure 3B**, Right Panel). In agreement with these findings, there was a parallel and significant reduction in the production of several proinflammatory cytokines such as TNF, IL-1β and IL-6 in animals treated with DCD as compared with those in the saline control group (**Figure 3D**). Similarly, DCD administration also led to a significant reduction in hepatic I/R-induced production of nitric oxide (NO, **Figure 3E**, Left Panel), which was associated with a significant attenuation of the expression of inducible nitric oxide synthase (iNOS, **Figure 3E**, Right Panel). Collectively, these finding suggest that DCD promotes significant protection against lethal hepatic I/R injury partly by attenuating I/R-induced local inflammatory responses.

### Intravenous DCD Administration Led to a Significant Inhibition of Lung Neutrophil Infiltration and Injury

**Figure 4:**
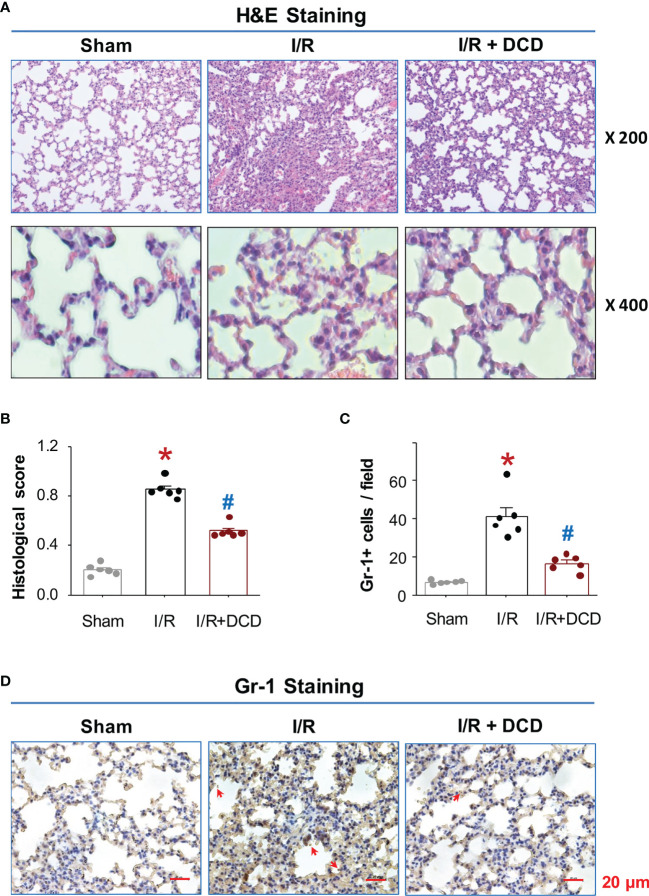
DCD protected mice against hepatic I/R-induced lung injury. **(A)** Histopathological characteristics of lung injury after hepatic I/R. Representative H&E histological images of lung sections at 24 h post the onset of reperfusion. Note a normal lung architecture in the “Sham” control, and extensive lung injury and neutrophil infiltration in the “I/R” group. DCD treatment group (“I/R + DCD”) exhibited a well-preserved tissue structure. **(B)** Histological Scores. Lung injury was assessed histologically using American Thoracic Society Documents’ lung injury scores (with skewed distribution), expressed as means ± SEM of 6 animals per group, and compared by non-parametric Kruskal-Wallis ANOVA test followed by the Dunn’s *post hoc* test. *P < 0.05 vs. “Sham”; ^#^ *P* < 0.05 vs. “I/R” group. **(C, D)** Lung neutrophil accumulation. Lung tissue sections were stained with Gr-1-specific antibodies, and the number of Gr-1-positive neutrophils (with normal distribution) per field was expressed as means ± SEM of 6 animals and compared by parametric one-way ANOVA followed by the Fisher Least Significant Difference (LSD) *post hoc* test **(C)**. *P < 0.05 vs. sham; ^#^ *P* < 0.05 vs. “I/R” group. Shown in Panel D were representative immunohistological staining of Gr-1-positive neutrophils (pointed by arrows) in lung sections.

It is well-known that hepatic I/R injury often causes remote tissue inflammatory injury, as characterized by the induction of a cascade of proinflammatory mediators that culminates in the recruitment of leukocytes to remote tissues (38–40). To further elucidate the mechanism underlying DCD-mediated protection, we assessed the effect of DCD administration on hepatic I/R-induced lung inflammatory injury. Consistent with previous findings (39), hepatic I/R induced marked lung inflammatory injuries as manifested by the increase in alveolar septal wall thickening, leukocyte infiltration, and alveolar congestion (**Figure 4A**). These hepatic I/R-elicited lung injuries were associated with a significant increase in histological lung injury scores (**Figure 4B**), as well as a parallel increase in lung neutrophil infiltration (**Figures 4C, D**). However, the hepatic I/R-elicited lung neutrophil infiltration and inflammatory injury was similarly and significantly inhibited by DCD administration (**Figures 4A–D**), suggesting that DCD conferred significant protection against lethal hepatic I/R by attenuating both local and remote inflammatory injuries.

### DCD Interacted With EGFR and Suppressed the Hepatic I/R-Induced EGFR Phosphorylation

**Figure 5:**
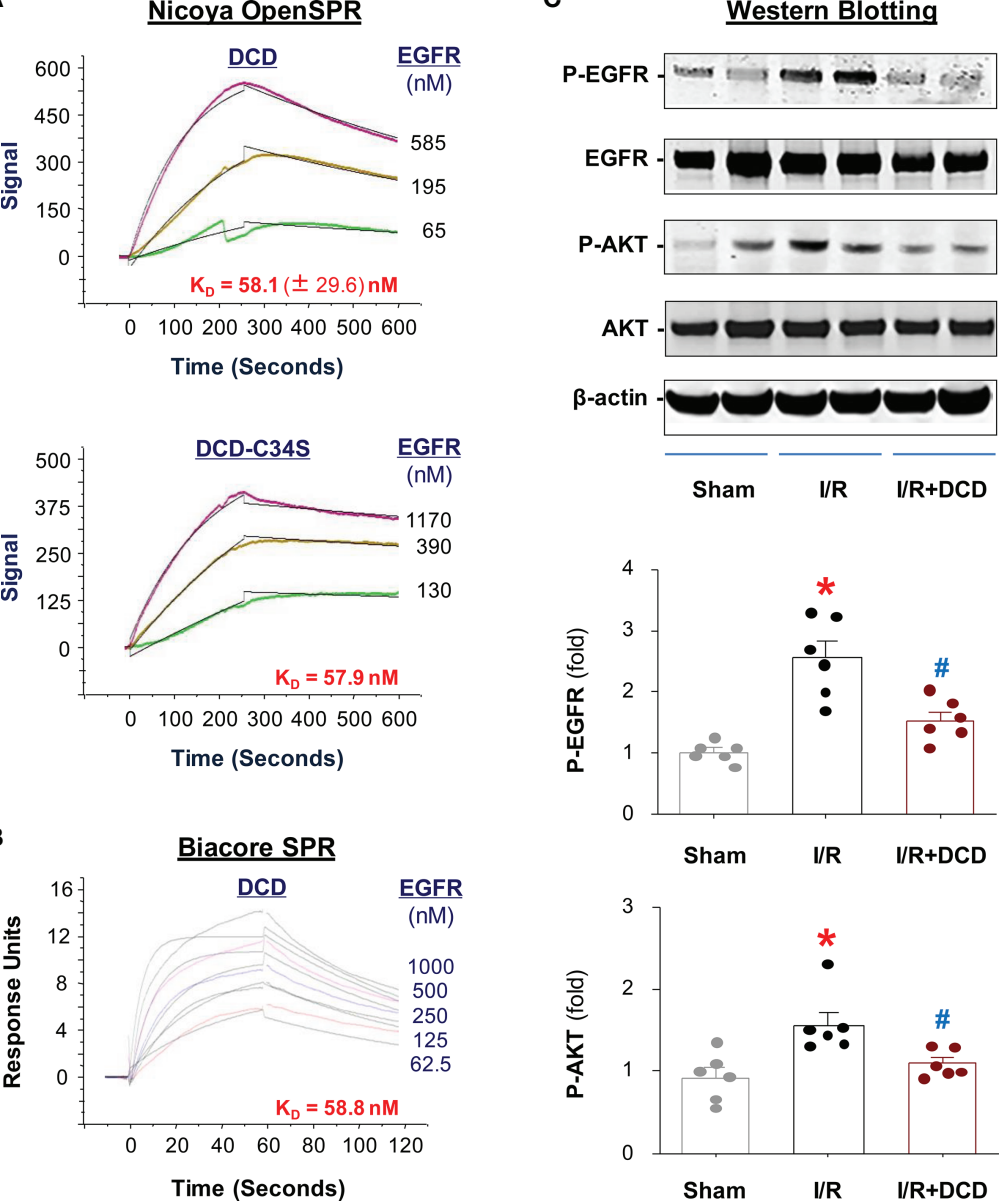
DCD interacted with EGFR and impaired I/R-induced EGFR phosphorylation in the liver. **(A)** Analysis of DCD-EGFR interaction using Nicoya Lifesciences OpenSPR. Highly purified DCD or DCD-C34S was immobilized on a NTA sensor chip, and recombinant protein corresponding to the extracellular domain of human EGFR (residue 25-645) was applied as analyte at various concentrations to estimate the dissociation equilibrium constant (K_D_). Shown in the graphs was the K_D_ (as mean ± SEM) of 1-3 independent experiments (n = 3, Upper Panel; n = 1, Lower Panel). **(B)** Analysis of DCD-EGFR interaction using Biacore SPR. Recombinant DCD was immobilized on CM5 chip, and the extracellular domain of human EGFR (residue 25-645) was applied as analyte at 5 different concentrations to estimate the equilibrium dissociation constant K_D_. **(C)** DCD administration attenuated hepatic I/R-induced phosphorylation of EGFR and AKT in the liver. Male C57BL/6 mice were subjected to hepatic ischemia and treated with control vehicle or DCD solution (0.5 mg/kg) at the beginning of the reperfusion. At 24 h post the onset of reperfusion, the liver tissue was subjected to Western blotting analysis of total and phosphorylated EGFR (“EGFR” and “P-EGFR”) and AKT (“AKT” and “P-AKT”), expressed as % of β-actin (with normal distribution), and compared by parametric one-way ANOVA followed by the Fisher Least Significant Difference (LSD) *post hoc* test. *P < 0.05 vs. “Sham”; ^#^ *P* < 0.05 vs. “I/R” group.

The epidermal growth factor receptor (EGFR) signaling has been suggested as a key regulator of the liver response to injury-elicited inflammation, as well as subsequent hepatocellular proliferation and neoplastic transformation (41). In an animal model of myocardial ischemia, the expression of EGFR in alveolar macrophages was up-regulated (42), and contributed to the expression of proinflammatory cytokines (such as TNF, IL-6, IL-1β), chemokines (such CXCL2/MIP-2, MCP-1, and CCL3) and iNOS (42). To gain further insight into the mechanisms by which DCD attenuated hepatic I/R-induced inflammation, we first tested the possibility that DCD interacted with the extracellular domain of EGFR using two Surface Plasmon Resonance (SPR) techniques. By using the Nicoya gold nanoparticle-based OpenSPR, we found that recombinant DCD exhibited a dose-dependent interaction with the extracellular domain of human EGFR (**Figure 5A**) with an equilibrium dissociation constant K_D_ of 58.1 ± 29.6 nM, as averaged from three independent experiments. In agreement with the minor difference between the side chains of residue 34 of DCD and DCD-C34S (i.e., “-SH group” versus “-OH group”, **Figure 1A**), these two proteins displayed almost identical binding affinities to EGFR (K_D_ = 58.1 nM for DCD versus K_D_ = 57.9 nM for DCD-C34S; **Figure 5A**). To further confirm this interaction, we also used the traditional Biacore SPR technique, and obtained an essentially similar K_D_ (58.8 nM, **Figure 5B**) for DCD-EGFR interaction, confirming that DCD and DCD-C34S analog interact with EGFR with similar affinities.

We then examined whether DCD administration affected hepatic I/R-induced phosphorylation of EGFR as well as a downstream signaling molecule, AKT, in the liver tissue. At 24 h post hepatic I/R, there was a significant increase in the phosphorylation of both EGFR and AKT (**Figure 5C**), although the total levels of EGFR or AKT were not obviously altered at this time point. However, intravenous administration of DCD resulted in a significant inhibition of hepatic I/R-induced phosphorylation of both EGFR and AKT, suggesting a possible role of EGFR signaling in the regulation of DCD-mediated anti-inflammatory actions.

### DCD Anti-Inflammatory Activity Required EGFR

**Figure 6:**
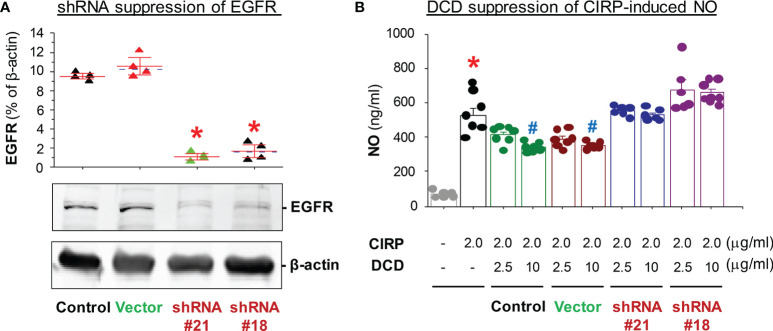
Knock down of EGFR expression impaired DCD-mediated suppression of CIRP-induced NO production in macrophage cultures. **(A)** Expression of EGFR in murine macrophage-like RAW 264.7 cells transfected with vector plasmid (“Vector”) or Egfr-specific shRNA-expression plasmid (“shRNA”). Lentivirus carrying vector plasmid (“Vector”) or Egfr-specific shRNA expression plasmid (“shRNA”) were used to transduce murine macrophage-like RAW 264.7 cells to produce stably transfected cell lines. The relative levels of EGFR in non-transfected controls, or macrophages transfected with vector plasmid (“Vector”) or Egfr-specific shRNA-expression plasmid (“shRNA”) was determined by Western blotting analysis, expressed as % of β-actin, and compared by parametric one-way ANOVA followed by the Fisher Least Significant Difference (LSD) *post hoc* test. *P < 0.05 vs. “Control”. **(B)** Effect of DCD on CIRP-induced NO production by macrophages transfected with different plasmids. Non-transfected RAW 264.7 cells (“Control”) or cells transfected with vector plasmid (“Vector”) or Egfr-specific shRNA (“shRNA #21” or “shRNA #18”) were stimulated with recombinant CIRP in the absence or presence of DCD at indicated concentration for 16 h, the level of NO in the culture medium was determined, and compared by parametric one-way ANOVA followed by the Fisher Least Significant Difference (LSD) *post hoc* test. *P < 0.05 vs. negative control (“-CIRP-DCD”); ^#^ *P* < 0.05 vs. positive control (“+CIRP” alone”).

To test the role of EGFR in the regulation of DCD-mediated anti-inflammatory actions, we transduced murine macrophage-like RAW 264.7 cells with lentivirus encoding either vector or Egfr-specific shRNA expression plasmids, and then compared the anti-inflammatory properties of DCD in these divergently transfected cells. In two lines of macrophages (shRNA#21 and shRNA#18) stably transduced by Egfr-specific shRNA plasmid, the constitutive expression level of EGFR was significantly attenuated (**Figure 6A**). Consistent with a previous report (22), recombinant mouse CIRP markedly stimulated macrophages to release nitric oxide (NO, **Figure 6B**), which was dose-dependently inhibited by the co-addition of DCD (**Figure 6B**). However, DCD failed to inhibit the CIRP-induced NO production in these Egfr-specific shRNA-expressing cells (**Figure 6B**), supporting a possible role of EGFR signaling in the regulation of DCD-mediated anti-inflammatory actions.

## Discussion

**Figure 7:**
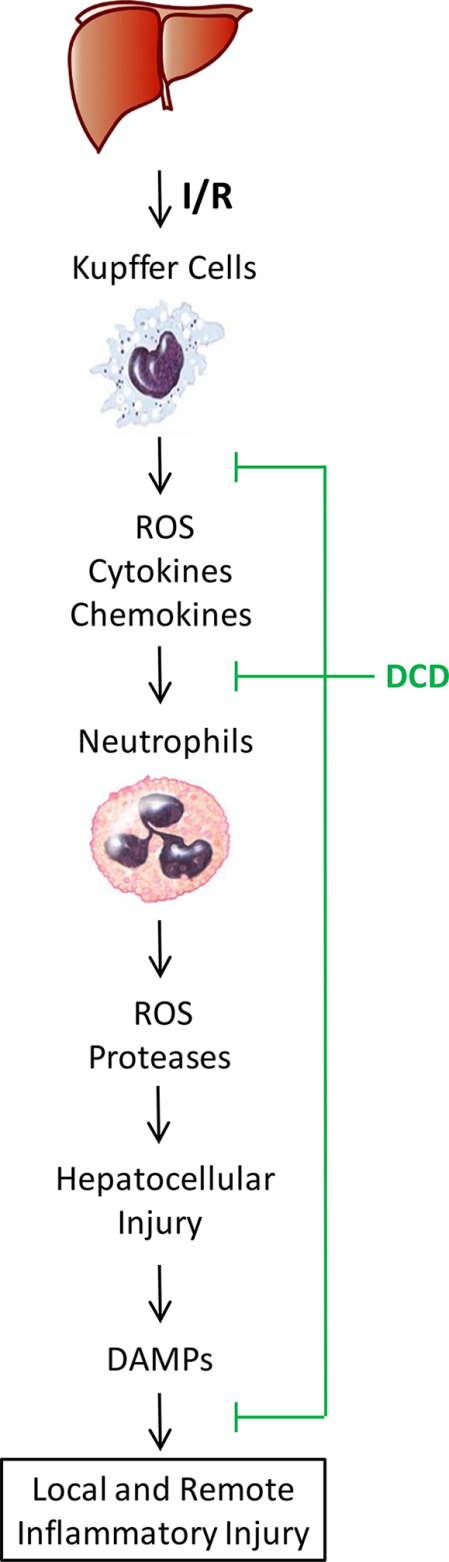
Proposed model for DCD-mediated protection against hepatic I/R injury. Hepatic I/R induces initial liver Kupffer cell activation and production of reactive oxygen species (ROS), chemokines, and cytokines that exert direct and moderate hepatic injury. These chemokines also facilitate the infiltration of neutrophils, which release more ROS and proteases to exacerbate a cascade of inflammatory injury, leading to hepatocellular death and passive release of DAMPs (such as CIRP and HMGB1) that further exacerbate a cascade of oxidative and inflammatory responses during a late stage of reperfusion. A sweat gland-derived peptide, DCD, can inhibit hepatic I/R-elicited chemokine expression, neutrophil infiltration, and associated inflammatory injury both locally in the liver and remotely in the lung. Our findings have suggested an exciting possibility to prevent hepatic I/R injury using DCD and analogs to suppress the phosphorylation of EGFR signaling, a key regulator of sterile liver inflammatory injury.

Hepatic I/R injury is an unavoidable consequence of major liver surgery and transplantation, and is mediated by sterile inflammatory responses jeopardizing the viability and function of multiple organs. It begins with initial hypoxic insult to ischemic tissues to cause moderate cellular damage (2–4), and continues with subsequent oxidative and inflammatory injury exacerbated by DAMPs such as CIRP (5) and HMGB1 (6, 7) during a late stage of reperfusion (1). Currently, there are no effective therapies to prevent hepatic I/R injury other than ischemic preconditioning and other preventive strategies (43). Thus, the primary objective of the current study was to explore the therapeutic efficacy and protective mechanisms of human DCD using a murine model of hepatic I/R injury. We demonstrated that treatment with recombinant DCD or a DCD-C34S analog conferred similar protection against lethal hepatic I/R, and concurrently attenuated hepatic I/R-elicited inflammatory injury both locally in the liver and remotely in the lung (**Figure 7**). This DCD-mediated protection was partly attributable to the attenuation of I/R-elicited neutrophil infiltration and inflammatory responses possibly through inhibiting I/R-induced EGFR signaling.

In response to hepatic I/R, liver resident Kupffer cells and infiltrated neutrophils orchestrate rigorous inflammatory responses manifested by the production of ROS, RNS, chemokines, and cytokines that collectively contribute to hepatic injury (1–3). Consequently, hepatocellular injury caused the passive release of DAMPs such as CIRP (5) and HMGB1 (6, 7), which further stimulate a feed-forward cycle of inflammatory injuries (8, 9) (**Figure 7**). In response to injury, a neutrophilic CXC chemokine, MIP-2, can be produced by macrophages and hepatocytes, and facilitate neutrophil recruitment and activation (44). Consistent with the role of CXC chemokines in mediating hepatic I/R-elicited deleterious inflammatory responses (45), we found a marked upregulation of Mip-2 expression in ischemic liver tissues. However, at the dose that conferred significant protection against lethal hepatic I/R, DCD also significantly attenuated hepatic I/R-elicited Mip-2 upregulation, and consequently reduced the infiltration of Gr-1-positive neutrophils into ischemic liver tissues. Collectively, these findings have suggested that DCD confers protection against hepatic I/R partly by attenuating neutrophil infiltration through inhibiting the expression of a key neutrophilic chemokine, MIP-2.

In agreement with the important contribution of neutrophil infiltration to I/R-elicited inflammatory IR injury (46), we observed a marked elevation of various proinflammatory cytokines (such as TNF, IL-1β, and IL-6) and reactive nitrogen species (NO) in the ischemic liver tissues. It has been shown that the inducible nitric oxide synthase (iNOS) responsible for the production of reactive nitrogen species (NO) is synergistically upregulated by various proinflammatory cytokines such TNF and IL-1β in the liver (47). Consistently, we found that DCD administration led to a parallel reduction of TNF, IL-1β, and NO, which was associated with a concurrent reduction of hepatic I/R-elicited iNOS upregulation. Our findings fully support the notion that excessive production of cytotoxic cytokines and NO may escalate severe liver injury (48), and suggest that the sweat gland-derived peptide DCD and its analogs could be developed to pharmacologically modulate injurious inflammatory responses.

It is known that hepatic I/R injury often causes remote tissue inflammatory injury as characterized by the induction of a cascade of proinflammatory mediators that culminates in the recruitment of leukocytes to remote tissues (38). Furthermore, the hepatic I/R-elicited remote tissue inflammatory injury and organ dysfunctions may similarly contribute to the lethal sequelae (38–40). Consistently, we found that hepatic I/R induced a marked lung inflammatory injury, as judged by the elevated neutrophil infiltration and pathological alterations of lung histology such as alveolar septal wall thickening, leukocyte infiltration, and alveolar congestion. However, the administration of DCD at the beginning of reperfusion resulted in a significant attenuation of the hepatic I/R-elicited lung inflammatory injury. Our findings that intravenous administration of DCD concurrently reduced liver and lung damage in both organs fully support the therapeutic potential of various anti-inflammatory agents in attenuating hepatic I/R-elicited multiple organ dysfunctions (43, 49, 50).

In addition, our current observations that DCD significantly attenuated hepatic I/R-elicited inflammatory response *in vivo* fully support our previous report that DCD differentially modulates the production of various cytokines/chemokines *in vitro* (22). Our present study also supports the notion that excessive release of DMAPs and excessive inflammation may further exacerbate the severity of hepatic I/R injury (51), although appropriate inflammatory responses might still be needed to facilitate tissue repair and promotes the re-establishment of homeostasis. The mechanism by which DCD suppresses inflammatory responses remains an exciting subject of future investigation. However, it is partly attributable to its possible inhibition of EGFR signaling, a key pathway implicated in the regulation of injury-elicited inflammatory responses in the liver (41, 42). First, Surface Plasmon Resonance analyses revealed that DCD could bind to the extracellular domain of EGFR with high affinities. Second, intravenous administration of DCD resulted in a significant suppression of hepatic I/R-elicited phosphorylation of EGFR, as well as a downstream kinase, AKT (35). Finally, the possible role of EGFR in the regulation of DCD-mediated anti-inflammatory activities was confirmed by knocking down the expression of EGFR by transfection with plasmids encoding for specific shRNA, which not only reduced Egfr expression, but also abrogated the DCD-mediated inhibition of NO production induced by a DAMP, CIRP. Collectively, these findings have suggested that DCD confers protection against lethal hepatic I/R partly through inhibiting EGFR signaling.

Our current study also has several obvious limitations. 1) We have not yet tried other routes of administration, and thus do not know whether DCD confers a similar protection if given *via* other routes of administration. 2) It is not yet known whether DCD administration affects hepatic I/R-induced expression of anti-inflammatory cytokines (e.g., IL-37 and IL-1Ra) in pre-clinical and clinical settings. 3) The intricate molecular mechanisms by which DCD divergently modulates the I/R-induced production of different cytokines and chemokines were not investigated in the present study. 4) It is presently not yet known whether genetically silencing EGFR would abrogate the activation and phosphorylation of various downstream signaling kinases (e.g., AKT and ERK) in murine macrophage cultures. In conclusion, our present study suggests that treatment with human DCD or DCD-C34S analog can potentially be developed as novel therapeutic strategies for hepatic I/R injury. The DCD-mediated protection was associated with a significant reduction in inflammatory injury both locally in the liver and remotely in the lung tissue, as manifested by the attenuation of neutrophil infiltration and production of proinflammatory cytokines. The anti-inflammatory action of DCD was partly dependent on its inhibition of EGFR signaling. Collectively, the present study suggests that the sweat gland-derived peptide DCD or its analogs might be developed as potential therapeutic agents to attenuate hepatic I/R-induced inflammation and tissue injury potentially by impairing EGFR signaling. Although it is not yet known whether our rodent model of acute hepatic I/R truly mimics human liver transplantation-associated I/R, we predict that DCD and DCD-C34S are likely protective against liver transplantation-associated I/R and inflammation in clinical settings.

## Data Availability Statement

The raw data supporting the conclusions of this article will be made available by the authors, without undue reservation.

## Ethics Statement

The animal study was reviewed and approved by the IACUC of the Feinstein Institutes for Medical Research.

## Author Contributions

XQ performed all animal experiments, and generated a rough draft of the manuscript. JL and KT generated recombinant DCD and DCD-C34S analog for the present study. MH and YA-A performed Biacore SPR analysis of DCD-EGFR interaction. SZ performed Open SPR analysis of DCD-EGFR interaction. WC was involved in some cellular experiments. PW supervised the animal study, interpreted some results, and together with MB, edited the manuscript. HW supervised the cellular and biochemical studies, interpreted most of the results, generated the final figures, significantly revised and finalized the manuscript. All authors read and approved the submitted version.

## Funding

This work was supported in part by the National Institutes of Health (NIH) grants R01GM063075 (to HW), R01AT005076 (to HW), R35GM118337 (to PW), and R01HL076179 (to PW). The Biacore T200 instrument was purchased with a NIH capital equipment grant (1S10RR033072 to YA-A).

## Conflict of Interest

PW and HW are co-inventors of a patent entitled “*Use of dermcidin in sterile anti-inflammatory conditions*” (US15/769,800). PW is a co-founder of TheraSource LLC, which has an option to license the relevant technology. MB is a part-time employee of TheraSource LLC.

The remaining authors declare that the research was conducted in the absence of any commercial or financial relationships that could be construed as a potential conflict of interest.

## Publisher’s Note

All claims expressed in this article are solely those of the authors and do not necessarily represent those of their affiliated organizations, or those of the publisher, the editors and the reviewers. Any product that may be evaluated in this article, or claim that may be made by its manufacturer, is not guaranteed or endorsed by the publisher.
